# A Bilobed Schwannoma in Roof of Orbit: A Rare Case Report

**DOI:** 10.1155/2012/139241

**Published:** 2012-04-01

**Authors:** Somya Dulani, Sachin Diagavane, Seema Lele, Harshal Gaurkhede

**Affiliations:** Department of Ophthalmology, JNMC Sawangi (M), DMIMS DU, Wardha, M-2 G-1, Meghdootam 442001, India

## Abstract

In this paper, we report a case of bilobed schwannoma, presented in the roof of orbit arising from supraorbital nerve. A 62-year male presented with a nontender mass in superior part of orbit and eccentric proptosis. Visual acuity and rest of ocular examination were normal. CT scan and MRI orbit revealed an extraconal homogenous bilobed mass, of size 3.5 to 2.5 cms in roof of orbit. Fine needle aspiration cytology was done, which was suggestive of schwannoma a peripheral nerve tumor. Successful surgical excision of intact bilobed schwannoma was done with careful separation and preservation of supraorbital nerve from which it was originated. Postoperative period was uneventful though rare, less than 1%, schwannoma can present as painless mass in the orbit and proptosis. Treatment of choice is surgical excision of intact tumor to prevent recurrence and preservation of peripheral nerve from which it arises.

## 1. Introduction

Schwannomas, also known as neurilemmomas, are benign peripheral nerve sheath tumors that present as slowly progressing, well-defined, unilateral orbital masses [1]. It accounts for 0.7% to 2.3% of all histopathologically proven orbital tumors. Peripheral tumors of the orbit arise from 3rd, 4th, 5th, and 6th cranial nerves and ciliary ganglion.

Morphologically, it is round to oval mass, sometimes causing bony indentation.

We report a case of bilobed schwannoma of supraorbital nerve presenting with painless mass in roof of orbit and eccentric proptosis. As per our knowledge a bilobed schwannoma has not been reported in literature till date. Radiological examination revealed an extraconal mass in the region of supraorbital notch and histopathological findings were typical of schwannoma. Total excision of tumor was done with preservation of nerve of origin. After about one and a half year there are no signs of recurrence of tumor.

## 2. Case Report

**Figure 1:**
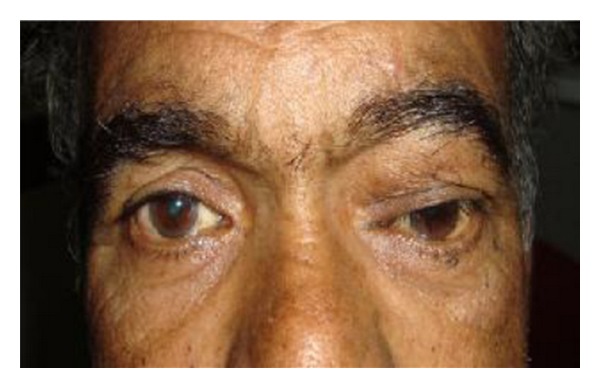
Clinical preoperative photograph of patient with upper lid mass and eccentric proptosis.

**Figure 2:**
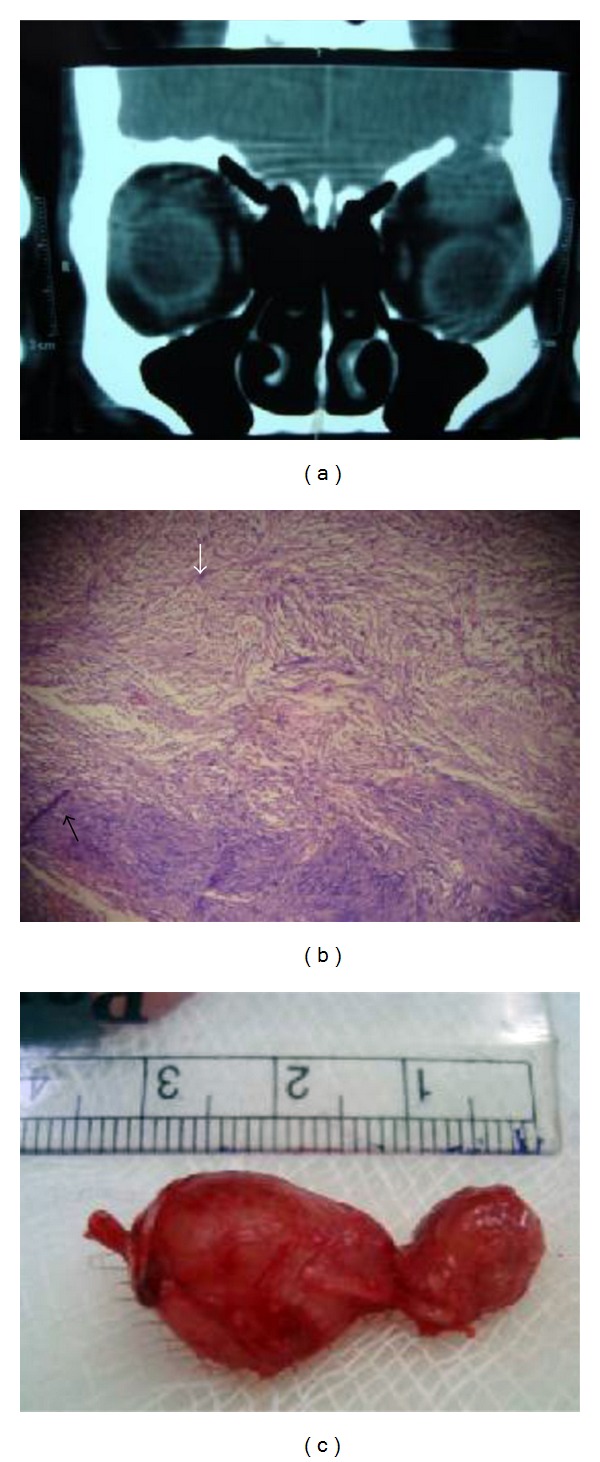
(a) Coronal CT reconstruction shows an extraconal mass causing inferior displacement of left globe with thinning of orbital roof. (b) Histopathological examination shows Antoni A cells (black arrow) and Antoni B cells (white arrow). (c) A bilobed excised tumor of size 3.5 cm to 2.5 cm with nerve of origin.

**Figure 3:**
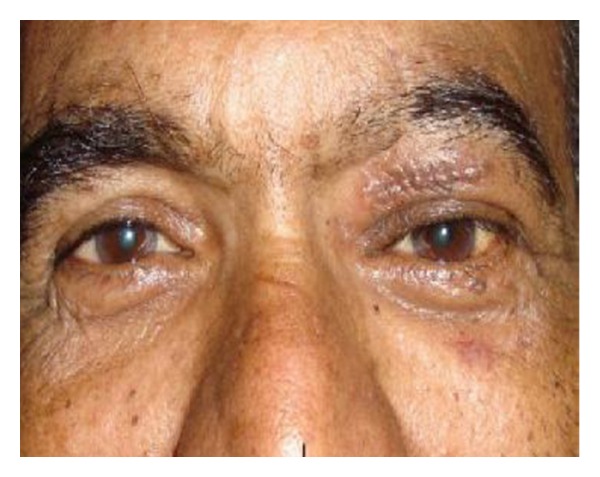
Postoperative photograph with sutures in situ.

 A 62-year-male presented with a nontender mass in upper lid above the medial canthus of left eye, with eccentric proptosis (Figure 1). He had history of swelling over the lid since past 2 years which was gradually increasing in size. On examination, the swelling was painless, firm to hard in consistency, nonpulsatile, nonreducible, and freely mobile. On ocular examination, the visual acuity of both eyes was 20/20 and ocular movements were normal. Corneal sensation was normal. Exophthalmometry revealed 3 mm proptosis in left eye. No abnormal findings were seen in anterior segment and fundus examination. Systemic evaluation was done to rule out the signs of neurofibromatosis. Haematological tests were within normal limits. CT-scan of left orbit revealed a smooth homogenous extraconal bilobed mass of size 3.5 to 2.5 centimeter in roof of orbit with thinning of superior orbital rim (Figure 2(a)). In MRI an isodense mass with respect to extraocular muscles was noted with probable diagnosis of a case schwannoma. Fine needle aspiration cytology findings were suggestive of schwannoma of peripheral nerve of orbit. Surgery was planned under general anesthesia. Through anterior orbitotomy, complete excision of tumor was done, with careful separation and preservation of nerve from which it was arising. During surgery a bilobed mass of size approximately same as shown by CT scan, that is, 2.5 cms to 3.5 cms was excised completely (Figure 2(c)). It was also noted that nerve from which tumor originated was supraorbital nerve due to its anatomical position near supraorbital notch. Postoperative period was uneventful (Figure 3), with a complaint of mild paraesthesia on left side of forehead. Histopathological examination of bilobed mass showed Antoni A cells and Verocays bodies confirming the diagnosis of schwannoma (Figure 2(b)). After one and a half year, no signs of recurrence or related complaints have been noted.

## 3. Discussion

Schwannoma generally occurs as an isolated tumor, however, in 2 to 18% of cases, it is associated with neurofibromatosis. Because schwannomas are encapsulated noninvasive tumors, it is important to differentiate these tumors from other masses with a similar presentation. Because masses more commonly arise from the supraorbital and supratrochlear branches, the patient may present with hypophthalmos with mild exophthalmos (2–4 mm).

Orbital schwannoma usually arises from sensory branches of ophthalmic division of the trigeminal nerve. It can present with exophthalmos as primary clinical symptom with limitation of mobility of eyeball [2].

The supraorbital and supratrochlear nerve is more commonly affected than the infraorbital nerve. Garg et al. [3] reported schwannoma in floor of orbit presenting with similar complaints but originating from infraorbital nerve and diagnosis was made on histopathological examination.

The nerve of origin is identifiable in about 32 to 47% of orbital schwannoma. The commonest presentation is painless, insidious proptosis [1]. Rarely, schwannomas may present with numbness in the distribution of the trigeminal nerve or with pain, or they may mimic the symptoms of sinusitis [2]. Grover et al. [4] presented a case of bilateral schwannoma [1] with its varied clinical presentation, preoperative investigations, operative findings, and appearance on light and electron microscopy. Although no single feature is pathognomonic, a multiplicity of clinical, radiographic, and surgical features point to this lesion. Two pathological types of cells are described in schwannoma, Antoni A and Antoni B cells. The nuclei of Antoni A cell palisade creates a picket fence type structure with interdigital cytoplasmic processes forming a pattern known as Verocays bodies [5].

In CT-scan schwannomas appear as smooth, ovoid, solitary orbital retrobulbar mass, most commonly in superior orbit with the long axis in the direction of the nerve, which is generally the anteroposterior direction [6]. MRI is isointense with respect to the extraocular muscle and cerebral gray matter on T1-weighted image and hyperintense on T2-weighted images.

Sometimes calcification is seen in peripheral nerve tumor which can be noted in CT [7]. Subramanian et al. [8] reported four cases of orbital schwannoma with cystic degeneration that presented with proptosis and decreased vision. CT scans showed a well-defined nonenhancing intraconal mass with cystic spaces. The histopathological examination was diagnostic for orbital schwannoma with cystic degeneration. Schwannoma should be included in the differential diagnosis of cystic orbital lesions. It may undergo cavitary changes which appear as a cystic mass with strawcolored fluid on gross pathological examination.

To prevent recurrence of tumor, various approaches for excision of tumor mass have been discussed. Frontoorbitozygomatic approach is also used for excision of supraorbital nerve schwannoma [9].

Although it is reported that intraorbital schwannoma accounts for 1–6% of all intraorbital tumors, the accurate diagnosis of the tumor origin may be difficult because of the complex orbital anatomy. Operative finding is the key to confirm the tumor origin [10]. In orbit most common sensory nerve of origin for schwannoma is supraorbital nerve, a branch of frontal nerve, presenting commonly as eccentric proptosis in downward and outward direction, but a bilobed schwannoma in roof of orbit is till now not reported in literature. If a schwannoma is managed properly by complete surgical excision of tumor, its prognosis is good and recurrence is not seen.
